# Excessive occupational heat exposure: a significant ergonomic challenge and health risk for current and future workers

**DOI:** 10.1186/2046-7648-3-14

**Published:** 2014-07-23

**Authors:** Rebekah A I Lucas, Yoram Epstein, Tord Kjellstrom

**Affiliations:** 1 Department of Public Health and Clinical Medicine, Centre for Global Health Research, Umeå University, Umeå 90187, Sweden; 2 Department of Physiology, Faculty of Medicine, Heller Institute of Medical Research, Sheba Medical center, Tel Hashomer, Tel Aviv University, Tel Aviv 6997801, Israel; 3 National Centre for Epidemiology and Population Health, Australian National University (ANU), Canberra 0200, Australia; 4 Institute for Global Health, University College London (UCL), London WC1E 6BT, UK

**Keywords:** Climate, Work, Productivity, Heat stress, Exposure, Occupational injury

## Abstract

Occupational heat exposure threatens the health of a worker not only when heat illness occurs but also when a worker’s performance and work capacity is impaired. Occupational contexts that involve hot and humid climatic conditions, heavy physical workloads and/or protective clothing create a strenuous and potentially dangerous thermal load for a worker. There are recognized heat prevention strategies and international thermal ergonomic standards to protect the worker. However, such standards have been developed largely in temperate western settings, and their validity and relevance is questionable for some geographical, cultural and socioeconomic contexts where the risk of excessive heat exposure can be high. There is evidence from low- and middle-income tropical countries that excessive heat exposure remains a significant issue for occupational health. Workers in these countries are likely to be at high risk of excessive heat exposure as they are densely populated, have large informal work sectors and are expected to experience substantial increases in temperature due to global climate change. The aim of this paper is to discuss current and future ergonomic risks associated with working in the heat as well as potential methods for maintaining the health and productivity of workers, particularly those most vulnerable to excessive heat exposure.

## Review

### Background

**Figure 1:**
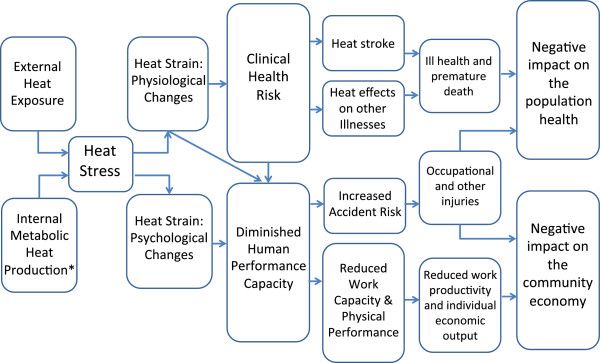
**A schematic summary of the proposed links between occupational heat exposure and health and productivity.** (Asterisk) Internal metabolic heat production significantly increases with physical movement or work.

Heat stress causes discomfort, increases physiological strain [1,2], decreases productivity and performance [3] and can increase accident rates [4] (Figure 1). Thus, understanding the effects and identifying the best means of reducing such impacts has been the focus of a considerable volume of research. The risks of excessive heat exposure have historically been well recognized in occupational settings such as in the military, mining and firefighting [5]. In hot low- and middle-income countries, the threat of excessive heat exposure is perhaps even greater on account of hot climatic conditions (at work and at home), limited resources or access to cooling methods (especially air-conditioning) and economic drivers to maintain productivity [3,6]. However, the prevalence or extent of excessive heat exposure in such occupational settings, countries and cultural contexts is not well appreciated. This results in poor implementation of appropriate and meaningful guidelines and heat management systems [7]. Added to this, climate change and increasing global temperatures will exacerbate occupational heat exposure in many places around the world [3].

The aim of this paper is to discuss current and future ergonomic risks associated with working in the heat as well as potential methods for maintaining the health and productivity of workers, particularly those most vulnerable to excessive heat exposure. To this end, the paper provides a summary of several issues: (a) the dangers associated with excessive occupational heat exposure, (b) mandatory protection or exposure to occupational heat, (c) self-regulated protection or exposure to occupational heat, (d) international standards concerning occupational heat stress and the applicability and relevance of such standards, (e) how workers and workplaces might adapt to reduce the impacts of excessive heat exposure and (f) as well as provide suggestions and future directions for practice and research.

### The dangers of occupational heat exposure

Humans have a tightly regulated internal body temperature range (approximately 37°C at rest) in which homeostatic processes are optimal (i.e. optimal structural and kinetic coordination of molecular, cellular and systemic processes). Thermoregulation mechanisms, including thermogenesis, autonomic (i.e. sweating and skin blood flow) and behavioural regulate this normothermic body temperature. Our behavioural adaptive capabilities are vast and paramount when managing thermal environments or reducing thermal loads [8]. Conversely, the effectiveness of our autonomic heat dissipation capacity is related to what type of clothing is worn and the environmental conditions. Indeed, our autonomic mechanisms can maintain a homeostatic internal body temperature within only a relatively narrow range of thermal environments [9,10]. Some occupational settings dictate a worker’s environment, clothing and behaviour as well as necessitate exposure to high thermal loads. Workers in such settings are at risk of suffering ill-health consequences due to excessive heat exposure. The difficulty with accurately determining which workers are most at risk of excessive occupational heat exposure is that heat tolerance varies broadly between individuals and even within an individual on a day-to-day basis. This is because environmental conditions, activity and individual biological factors can shift and change to escalate the risk of occupational heat exposure.

#### Environment

##### Climate

Climate conditions dictate the efficacy of autonomic heat loss mechanisms as these mechanisms rely on the temperature and water vapour pressure gradients between the body’s surface and the environment [11,12]. Climatic thermal balance points represent the minimum bodily thermal gradient compatible with the transfer of the metabolic heat to the skin without inducing undue strain on the circulatory system (i.e. reduced cardiac filling pressure and stroke volume, elevated heart rate) [9,13]. Therefore, for humans, a *thermal extreme* can be defined as the upper limit of humans’ ability to maintain thermal balance and a steady-state internal body temperature [14]. Hot and humid climatic conditions create a thermal heat extreme as heat loss from the body to the environment becomes increasingly difficult and an ‘uncompensable heat situation’ can easily develop whereupon internal body temperature necessarily rises irrepressibly.

##### Climate change

Climate change is heating the earth’s surface, with world-average temperatures conservatively forecasted to increase within the range of 1.1°C to 4.8°C by 2100 (under Representative Concentration Pathways (RCP) scenarios 4.5, 6.0 and 8.5 [15]). Furthermore, extreme climate events are predicted to increase in intensity, duration and frequency in the future [16]. A direct effect of climate change is increased ambient heat exposure, particularly in tropical countries where heat exposure levels are already verging on untenable during parts of the day. For example, recent estimates for Thailand and Cambodia indicate that in 2050 during the hottest month of the year, it will be too hot to work safely outdoors and perform heavy labour for at least half of the working day (40%–60% of current working hours lost) [17]. Such impacts have obvious ramifications for production and productivity in vulnerable regions. Indeed, climate change has significant ramifications for workplace health and productivity as temperature increases alone are anticipated to disrupted production processes in nature (agriculture, forests and fisheries) and impair work capacity in climate-sensitive occupations (e.g. agriculture, construction or non-air conditioned workplaces) [3,18].

##### Geographical

**Figure 2:**
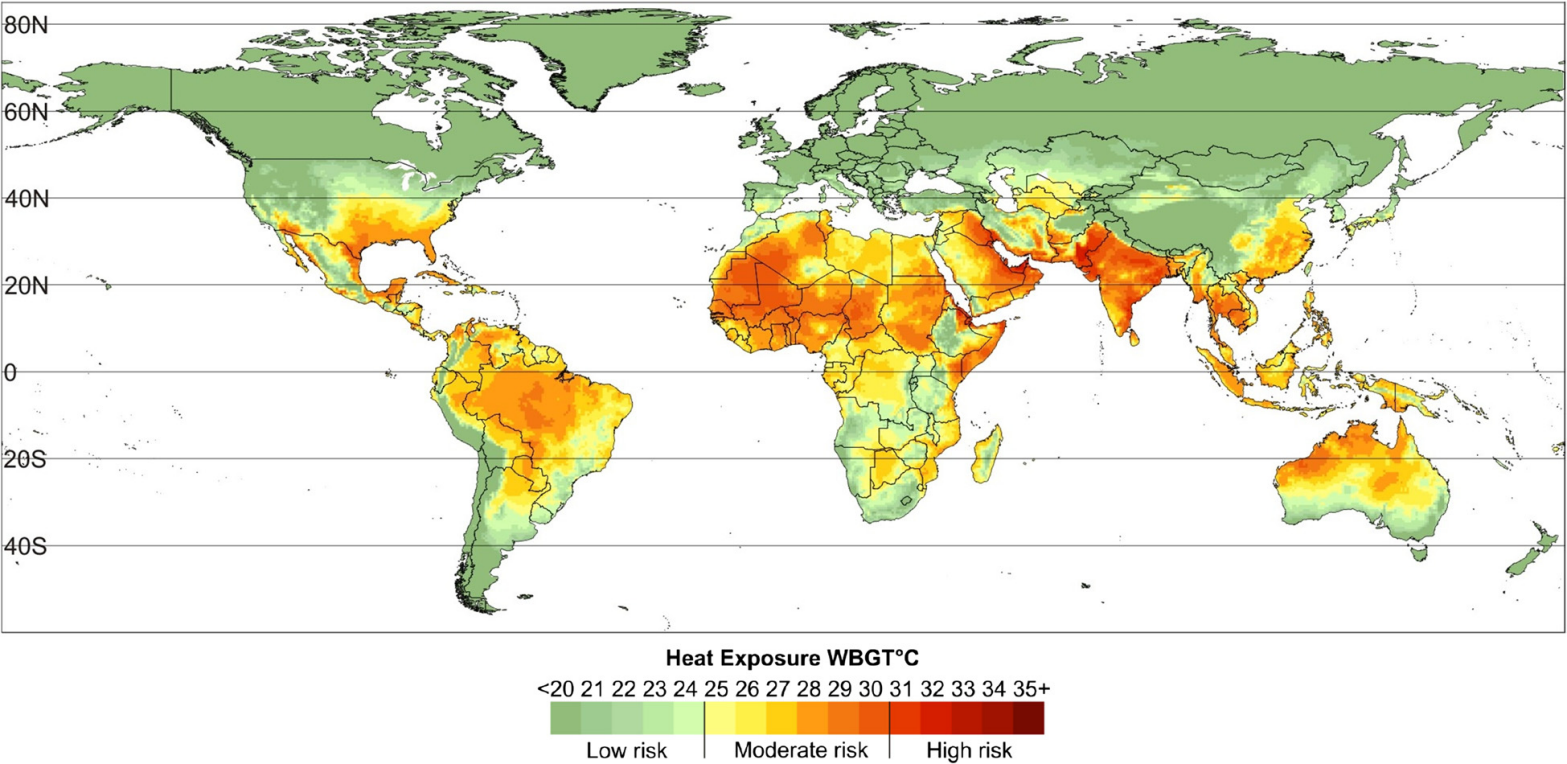
**Thirty-year average (1980–2009) of monthly average wet bulb globe temperature (WBGT).** In the afternoons indoors or in full shade for the hottest month (e.g. August for USA and Egypt, April for India, January for Australia) in each part of the world (0.5 × 0.5° grid cells). The yellow regions (WBGT 25–27) indicate where workers performing heavy labour are affected by hot climate conditions; the orange (WBGT 28–30) and red (WBGT 31 +) regions indicate where workers performing moderate or light work are affected (Hyatt et al. unpublished).

Subtropical and tropical countries routinely experience high climatic temperatures, often in conjunction with high humidity levels. Rapid urbanization and the associated urban heat island effect also substantially increases local temperatures and reduces the temperature drop at night [19,20]. Thus, heat exposure poses more of an occupational risk for workers in subtropical and tropical regions (Figure 2), particularly those in cities and urban settings.

##### Sociocultural context

Behavioural thermoregulation is our most powerful means of removing or reducing heat exposure and the prospect of heat illness. Subsequently, circumstances that inhibit behavioural adaptations (e.g. mandatory uniform or protective clothing, payment per output or lack of employment alternative) can significantly increase a worker’s susceptibility to the risks of excessive heat exposure. Institutional environments, such as the military, can determine an individual’s exposure to excessive heat and influence their heat tolerance (i.e. mandatory uniforms/protective clothing, acclimatization/training). Subsequently, heat-related guidelines, cooling equipment and training methods are well established in such organizations [21]. Yet, heat illness remains a serious concern, as highly motivated individuals can exert themselves beyond safe thermal limits, sometimes to very serious health consequences [22,23]. The social norms or culture of an institution can certainly cultivate such motivated individuals. For workers with a low socio-economic status, payment per output or fear of losing employment can determine that workers drive themselves beyond safe thermal limits [24].

#### Actions

##### Exertional

In humans, a large amount of heat is released when energy is consumed (via adenosine triphosphate) for cellular processes such as membrane transport, chemical reactions and mechanical work. During exercise, internal body temperature increases in proportion to workload/metabolic rate [9,25,26]. When the heat generated from muscular work cannot be adequately dissipated by heat loss mechanisms, body temperature increases. This can be a safe and beneficial process as it triggers positive cardiovascular and cellular adaptations (i.e. heat shock proteins) that can improve thermal tolerance [27]. Indeed, acute increases in internal body temperate can safely be withstood (i.e. +40°C in competitive elite athletes), presuming that appropriate training (or acclimation) and recovery is ensured. Yet still, the risk of exertional heat illness (including heat cramps, heat syncope, exercise exhaustion, exertional heat stroke and possibly exertional hyponatremia) remains when working or exercising in hot, humid conditions, wherein an uncompensable heat situation can easily develop that initiates negative health consequences [28,29]. Even in the military, where the combined hazards of a hot climate and physical exertion are well recognized and detailed regulations/practices are followed, exertional heat illness continues to be a problem during training and operations [30]. For example, the Israeli Defence Forces reported 150 heat illness cases between 1988–1996 [22], whereas the USA military saw 5,246 army soldiers hospitalized for heat illness between 1980–2002 [31] and 1,060 heat injury events occurred in Iraq/Afghanistan from 2008 to 2012 [30].

##### Protective clothing

Protective clothing can create a serious heat stress problem, as it can have no or low moisture permeability and high insulating properties. Such properties inhibit sweat evaporation and normal heat dissipation, increasing internal body and skin temperatures and causing excessive sweating [32-34]. Protective clothing (both impermeable or semipermeable) also often adds bulk and weight, particularly if a closed circuit breathing apparatus is included [34]. Thus, protective clothing increases the metabolic cost and thermal load of performing a task. For example, at a low work intensity (30% of maximal work capacity), wearing firefighter protective clothing and breathing apparatus can reduce exercise tolerance by 84% [32,33]. Indeed, the thermal challenge associated with working in protective garments is well appreciated for firefighters, chemical industries and the military [11,33,35].

In reality, clothing of any nature creates a barrier for heat and vapour transport between the skin and the environment [35]. For example, in India, women construction workers wear polyester shirts over their traditional sari for modesty reasons. This practice traps the sari beneath a less permeable fibre decreasing air movement, vapour permeability and increasing the clothing’s insulation [36]. Such clothing practices create a higher heat load for these women. Thus, it is important that the fundamental aspects of clothing properties and thermoregulation are understood and appropriately managed in all occupational settings frequently exposed to high thermal loads.

#### Who is at risk?

##### Personal risk factors

At an individual level, a single predisposing risk factor may reduce an individual’s heat tolerance, while a combination of several factors synergistically increases the risk of heat illness [37-39]. Such personal risk factors include low physical fitness, lack of acclimation, surface-to-mass ratio, age, fatigue, prior heat illness or dehydration [37,39]. Added to this, some disease states (i.e. cardiovascular disease, diabetes mellitus or infectious diseases) or medications can impede thermal tolerance as well as drug abuse or alcohol [37,40]. Thus, an individual’s risk of becoming heat intolerant can vary on a day-to-day basis or slowly change according to chronic influences.

##### Global health trends

The general global population is increasingly sedentary, ageing, with higher rates of non-communicable diseases [41]. Therefore, the general working population is increasingly less fit, older, with a higher prevalence of chronic disease and medication use. Such a combination of personal risk factors reduce thermal tolerance of the average worker and increase their susceptibility to heat-related illness, on a global scale. Continuation of these global health trends has severe ramifications for general and occupational health and will likely increase heat-related illness and mortality [40].

### Mandatory protection or exposure to occupational heat

There are certain occupations or work circumstances where high heat exposure cannot be avoided. Given the known risks and decrements to work performance and health, it is unsurprising that extensive heat prevention procedures and strategies have been investigated and implemented in such settings to better manage heat strain and reduce the risk of serious heat illness. These heat prevention strategies include: identifying high-risk individuals, heat acclimation, exercise/rest guidelines, fluid and electrolyte replacement and vigilance [31,42-44]. The benefits of an institutionalized work environment are that such regulations and practices can be enforced and regulated, not only by the organization (i.e. by employers, supervisors or senior officers) but also by external organizations (i.e. local, national or government health and safety authorities). Furthermore, there is a formal opportunity for educating individuals as to the risks associated with heat exposure, appropriate preventative actions as well as recognizing the signs and symptoms of heat illness. Though to date, there is no evidence indicating whether such education reduces the incidences of heat illness or improves workplace performance.

Some institutions may require individuals to be medically cleared prior to employment or active service. In such instances, medical standards are used to determine an individual’s suitability for employment and related duties [45]. The obvious benefits of these measures are that individuals are medically tested and cleared for the rigors of their training/work. Subsequently, individuals susceptible to the risks of excessive heat exposure can be identified and removed from an unsafe environment. Though, their employment opportunity is reduced in a regulated and health-insured job sector. Another disadvantage is that medical standards are only as good as the clinical and scientific input underlying them.

It is also important to consider if heat prevention regulations can become too controlled or normalized as well as what drives or underlines such regulations. Are regulations truly protecting the worker or more so the employer in our increasingly litigious society? Out of necessity, heat exposure regulations have inbuilt safety margins to protect the majority (i.e. 90%). This determines that regulations are stricter than needs be for a large percentage of workers and subsequently might unnecessarily constrain an individual and reduce their work rate. In view of this, subjective feelings of thermal sensation and comfort might best indicate a worker’s level of heat stress and thus avoid unnecessary restraint or risk for that individual. Certainly, subjective feelings of thermal sensation and comfort integrates feedback from the skin and the body’s core and can drive thermoregulatory behaviour if allowed [46]. Thus, perceptual awareness in conjunction with autonomous control over work conditions, work rates and work limits might be the most valid, sophisticated and cheapest means of determining heat exposure limits at an individual level. However, reliance on such psychophysiological indicators of heat stress/strain (instead of regimented heat prevention methods) presumes that an individual can always be trusted to heed signals from their own body and behave accordingly. Also, mandatory heat prevention strategies may be required to successfully complete the task. For example, in the military, mandatory work/rest cycles are essential for enduring sustained missions where solders are required to operate as a unit.

### Self-regulated protection or exposure to occupational heat

Self-pacing and rest breaks are autonomous safeguards intuitively activated to manage thermally stressful conditions and reduce heat strain [47-49]. Such actions attenuate increases in internal body temperature, reduce fatigue, maximize long-term endurance and enable sustained activity over the workday [49-51]. Indeed, traditional cultural practices (e.g. siesta, reduced work intensity, large hats) have been effective strategies for workers to self-regulate and protect themselves from excessive heat exposure in the past [11]. However, self-pacing can reduce work rate and production [3,49]. Therefore, in some settings, workers will either achieve less or work longer (enduring longer periods of heat exposure) to meet their quota [6,52]. Notably, mandatory work/rest cycles may also reduce work rate and given inbuilt safety margins (as mentioned prior); mandatory regimes would presumably cause a larger reduction in individual work rate than self-paced practices, although this has not been examined in an occupational setting.

High rates of heat illness have been reported in some occupational sectors where heat exposure and heat prevention measure are not formally regulated (such as agriculture). For example, from 1992–2006, 68 crop workers in the USA died from heat stroke, representing a rate nearly 20 times greater than that of all US civilian workers (with a mortality rate of 0.4 per 100,000 workers as compared to 0.02 for all US civilian workers) [23]. By way of comparison, 37 heat illness-related deaths were reported in the US Army from 1980 to 2002 (representing a mortality rate of 0.3 per 100,000 soldiers) [31], thus indicating that appropriate heat management programs and policies can reduce the risks of occupational heat exposure. Also, income and livelihood are pervasive motivating factors that can drive workers to ignore psychophysiological indicators of heat strain. For example, there is strong causal evidence that repeated heat exposure, dehydration or volume depletion and strenuous work in tropical climates are key risk factors or essential co-factors in the development of the Mesoamerican nephropathy epidemic [24,53]. Therefore, whether self-paced or regulated work/rest cycles are implemented, it is vital that workers are appropriately compensated for the work they perform and not penalized for environmental constraints.

### What regulations are established, and why/how are they set?

The International Organization for Standardization (ISO) since 1947 has facilitated international coordination and unification of industrial standards. There are collections of ISO standards concerned with the ergonomics of the thermal environment that specify appropriate protective measures and good practices when working in a hot, moderate and cold environments [11]. These standards have principally been designed and developed in accordance with data from Europe and the USA [54]. Subsequently, there has been some debate regarding the validity, ambiguity and usability of such standards in industrially developing countries, as differences in physiology, anthropometrics and culture may determine that ISO standards are unrealistic or unreasonable to enforce in different work settings [54].

Heat stress indices included in ISO standards have been developed to predict the physiological strain from a stressful environmental condition. Such indexes give a single number representative of the interaction between the basic climate parameters (air temperature, air humidity, air movement over the skin (wind speed) and heat radiation (i.e. from the sun), which can then be linked to a corresponding physiological strain and subsequently, be used to design or establish safe work practices, work limits and work conditions [55,56]. Numerous heat stress indexes for workplace application have been published in the last century (the first was published in 1905) including Wet Bulb Globe Temperature (WBGT), created in the USA in the 1950s; Predicted Heat Strain model (PHS), incorporated into ISO 9886 and subsequently developed further [57]; thermal work limit (TWL), created in Australia in 2002 [58]; Universal Thermal Climate Index (UTCI), established by the European Union and WMO in 2009 [59]; Humidex, used in Canada and many others (Epstein and Moran 2006). WBGT is the most widely used for workplace heat stress assessments [55] and is the basis for an international standard [42] and many national standards or guidelines (e.g. [60]), though there are limitations with the WBGT, including its underestimation of the stress of restrictive evaporation and responses to air movement [61]. The WBGT index is also calculated purely from environmental variables and therefore the effects of metabolic heat production and clothing are not included in its scope [62]. The additional application of ISO standards (such as ISO 7243) provides WBGT reference values for a variety of environmental and personal conditions (i.e. clothing and workload) [62]. However, these reference values are valid only for the metabolic and clothing parameters defined [62]. Furthermore, similarly, the UTCI, though validated for all relevant combinations of climate parameters, incorporates one metabolic workload (corresponding to walking 4 km h^−1^) and only seasonal European clothing. Knowing such limitations is essential in the appropriate application of any heat stress index. *For further information on the limitation and variety of heat stress indexes, please refer to*[11,56,61,63-65]*.*

It is important to pay due attention to the fact that both ISO standards and heat stress indexes are guidelines and tools designed to provide an *estimate* of the relative thermal risk and the appropriate action. They are based off norms (albeit it typically western norms) and subsequently cannot and perhaps should not be expected to encompass all people in every situation. It can also be argued that ISO standards are by design conservative, with an inbuilt safety margin. However, such standards play an important role in protecting workers and providing a framework with respect to appropriate work conditions. How such standards and heat indexes can be used in specific geographical and socioeconomic contexts requires further investigation. For example, how the *informal* work sector receives and applies information and guidance regarding such workplace standards needs consideration, particularly in regions with a large informal work force.

### Can we adapt? Do we want to? (Adaptation or maladaptation?)

Physiologically, humans adapt to heat by increasing the efficacy of physiological heat loss mechanisms and increasing their cardiovascular capacity [44,66]. Such adaptations reduce heat strain and improve physical performance in the heat. Heat acclimation (via a hyperthermic exercise intervention) is virtually completed within 14 days [67] determining that physiological heat adaptation can be maximized relatively quickly if appropriate action is taken. Subsequently, though workers at risk of excessive heat exposure certainly benefit from heat acclimation [44], their physiological adaptive capacity is limited and, thus, excessive heat exposure remains a risk [31]. Notably, previous studies have largely focused on heat acclimation and the physiological adaptation achieved in a climatic chamber as opposed to natural and prolonged heat acclimatization. Therefore, it remains uncertain if workers’ or soldiers’ exposure to hot climatic conditions over prolonged periods (i.e. months or years) might physiologically adapt further. However, it is likely that any such adaptation (if evident) would be small when compared to other thermoregulatory modulators (e.g. physical fitness, disease state, medication use).

There are technical and behavioural modifications and adaptations to counter the negative effects of excessive heat exposure in working populations. In the short-term, appropriate work conditions and interventions to alleviate heat strain (i.e. easy safe access to water and toilet facilities, regimented rest/drink breaks, appropriate clothing, personal cooling techniques and equipment, payment per hour versus payment per output) could significantly improve workers health as well as aid production and productivity in parts of the world [6,43]. In the long-term, changes to building and urban design would help mitigate the impacts of increasing global temperatures and improve work and living conditions worldwide [68,69]. Such interventions and innovations could also help alleviate reliance on air-conditioning, which as a technical solution and ‘easy fix’ is fraught with difficulties as it exacerbates electricity consumption, the urban heat island and climate change itself [70]. However, such adaptations and countermeasures will not be undertaken if the magnitude of the problem is not understood. Also, as mentioned earlier, some behavioural adaptations such as self-pacing and work/rest ratios can reduce production and productivity [3]. If heat management regimes interfere with an individual’s ability to carry out daily tasks, such as work or household chores, such heat adaptations have arguably become maladaptation. If this is the case, climate change mitigation and sustainable methods of reducing heat exposure are imperative. It is worth noting that eliminating all forms of heat exposure removes the stimulus for acclimatization and acclimation, which as discussed earlier, significantly affects heat tolerance. Thus, while every effort should be made to mitigate increasing in global temperatures (for a plethora of environmental, economic, social and health reasons), safe heat exposure with appropriate recovery is beneficial to workers and the general population’s health. That being said, we must remind ourselves that some workers and populations around the world live in increasing hot environments with little respite or relief.

## Conclusions

### Suggestions and future directions for practice and research

Any reduction in capacity to perform daily activities due to heat, cold or extreme weather should be considered a ‘health effect’ of climate conditions in light of the WHO’s definition of health (‘Health is a state of complete physical, mental and social well-being and not merely the absence of disease or infirmity’) [71]. Thus, occupational heat exposure threatens the health of a worker not only when heat illness occurs but also when productivity is undermined. It is imperative that such a definition of ‘health effect’ be applied if the true magnitude of excessive workplace heat exposure is to be understood.

Workers in low- and middle-income tropical countries are likely at highest risk of excessive heat exposure as these countries are densely populated, have large informal work sectors and are expected to show substantial temperature increases due to global climate change. Further research identifying the current risks and impacts of occupational heat exposure is vital for comprehensive climate impact assessments. Such research could have an important role in driving policy with respect to climate change adaptation and mitigation and therefore holds significance not only for current but also for future working populations. Also, research concerning occupational heat exposure and health inequities needs to be undertaken for evidence-based policy advocacy regarding work conditions in different parts of the world.

To minimize excessive heat exposure in the workplace, it is recommended that workers and employers regularly review the potential impacts of heat on workers’ health and productivity. From such information, workers and employers can adopt the most effective heat prevention strategy and enable intelligent and safe work practice.

Heat-related work capacity losses are an important justification for more active climate change mitigation policies and programs all around the world. Due attention, analysis and directives need to be taken in response to this climate change and health challenge. Any program attempting to address health issues associated with climate conditions should consider workplace heat exposure.

## Abbreviations

ISO: International Organization for Standardization; PHS: Predicted Heat Strain model; RCP: representative concentration pathways; TWL: thermal work limit; UTCI: Universal Thermal Climate Index; WBGT: Wet Bulb Globe Temperature.

## Competing interests

The authors declare that they have no competing interests.

## Authors’ contributions

RAIL participated in the conception and outline, article retrieval and review, manuscript writing and final approval. YE participated in the article retrieval and review, critical revision and final approval. TK participated in the conception and outline, article retrieval and review, critical revision and final approval. All authors read and approved the final manuscript.
